# Interferon γ-Induced Nuclear Interleukin-33 Potentiates the Release of Esophageal Epithelial Derived Cytokines

**DOI:** 10.1371/journal.pone.0151701

**Published:** 2016-03-17

**Authors:** Jing Shan, Tadayuki Oshima, Liping Wu, Hirokazu Fukui, Jiro Watari, Hiroto Miwa

**Affiliations:** 1 Division of Gastroenterology, Department of Internal Medicine, Hyogo College of Medicine, Nishinomiya, Japan; 2 Department of Gastroenterology, The Third People's Hospital of Chengdu, Chengdu, China; University of Leuven, Rega Institute, BELGIUM

## Abstract

**Background:**

Esophageal epithelial cells are an initiating cell type in esophageal inflammation, playing an essential role in the pathogenesis of gastroesophageal reflux disease (GERD). A new tissue-derived cytokine, interleukin-33 (IL-33), has been shown to be upregulated in esophageal epithelial cell nuclei in GERD, taking part in mucosal inflammation. Here, inflammatory cytokines secreted by esophageal epithelial cells, and their regulation by IL-33, were investigated.

**Methods:**

In an *in vitro* stratified squamous epithelial model, IL-33 expression was examined using quantitative RT-PCR, western blot, ELISA, and immunofluorescence. Epithelial cell secreted inflammatory cytokines were examined using multiplex flow immunoassay. IL-33 was knocked down with small interfering RNA (siRNA) in normal human esophageal epithelial cells (HEECs). Pharmacological inhibitors and signal transducers and activators of transcription 1 (STAT1) siRNA were used to explore the signaling pathways.

**Results:**

Interferon (IFN)γ treatment upregulated nuclear IL-33 in HEECs. Furthermore, HEECs can produce various inflammatory cytokines, such as IL-6, IL-8, monocyte chemoattractant protein 1 (MCP-1), regulated on activation normal T-cell expressed and presumably secreted (RANTES), and granulocyte-macrophage colony-stimulating factor (GM-CSF) in response to IFNγ. Nuclear, but not exogenous IL-33, amplified IFN induction of these cytokines. P38 mitogen-activated protein kinase (MAPK) and janus protein tyrosine kinases (JAK)/STAT1 were the common signaling pathways of IFNγ-mediated induction of IL-33 and other cytokines.

**Conclusions:**

Esophageal epithelial cells can actively participate in GERD pathogenesis through the production of various cytokines, and epithelial-derived IL-33 might play a central role in the production of these cytokines.

## Introduction

For decades, the esophageal epithelium was thought to be a tissue that forms a barrier against caustic chemical injury, remaining quiescent until activated by an invading army of immune effector cells. However, this concept has been challenged by studies showing that esophageal epithelial cells can produce various inflammatory cytokines, such as interleukin (IL)-8 and IL-6, in response to intraluminal stimuli including acid, bile acid, and trypsin [1–6]. Since esophageal inflammation occurs prior to macroscopic or even microscopic signs of mucosal injury in gastroesophageal reflux disease (GERD), a new view has been presented that suggests that intraluminal reflux stimuli cause cytokine-mediated mucosal injury rather than being directly mediated by caustic acid [7]. Esophageal epithelial cells, the first site of exposure to various intraluminal stimuli, are likely to serve as the initiating cell type in esophageal inflammation, and play an essential role in the pathogenesis of GERD. Studies exploring the expression profile of inflammatory cytokines in GERD have demonstrated that epithelial cells secrete IL-8 and IL-6. Additionally, interferon gamma (IFNγ), tumor necrosis factor alpha (TNF-α), IL-1β, IL-10, monocyte chemoattractant protein 1 (MCP-1), and regulated on activation normal T-cell expressed and presumably secreted (RANTES) have been found to be upregulated in mucosal biopsy specimens [1, 8, 9]. However, whether these inflammatory cytokines can be secreted by esophageal epithelial cells and how they are regulated remains unclear.

IL-33 is a new tissue-derived cytokine that is a novel member of the IL-1 cytokine family. IL-33 is constitutively expressed in endothelial and epithelial cells of tissues exposed to the environment [10]. IL-33 appears to be a cytokine that acts as an intracellular nuclear factor with transcriptional regulatory properties [11, 12]. Epithelial-derived IL-33 is a critical regulator of both innate and adaptive immunity. IL-33 participates in many acute and chronic inflammatory gastrointestinal diseases, such as ulcerative colitis, and gastritis [13, 14]. Exogenous IL-33 can be upregulated by proinflammatory cytokines, such as IFNγ and TNF-α, in different cell types [15–17]. We have recently reported that the expression of IL-33 was upregulated in esophageal epithelial cells in reflux esophagitis. IFNγ upregulated nuclear IL-33 in an esophageal stratified squamous epithelial model, while IFNγ-induction of IL-8 and IL-6 was IL-33 dependent [18]. However, the role IFNγ and epithelial derived-IL-33 in regulating other inflammatory cytokines found in GERD, and the underling signaling pathways involved have not been investigated.

Therefore, in the present study, we used a three-dimensional stratified squamous epithelial model using normal human esophageal epithelial cells (HEECs) [19–21] to investigate the production and regulation of IL-33 and inflammatory cytokines associated with GERD, and the underling signaling pathways. IL-33 knockdown by small interfering RNA (siRNA) was used to explore the role of IL-33 in IFNγ-induced cytokine production in esophageal epithelial cells.

## Materials and Methods

### Cell culture

HEECs were purchased from ScienCell^™^ Research Laboratories (Carlsbad, CA), and were primary human esophageal cells. The batch we used in this study was derived from fetus (21 weeks, female). For air-liquid interface (ALI) culture, Transwell^™^-Clear wells (Costar Co., Cambridge, MA) were coated with collagen, human fibronectin and BSA. The cells were cultured in epithelial cell medium-2 (EpiCM-2, ScienCell^™^ Research Laboratories) and subcultured to Transwell^™^-Clear wells until approximately 80% confluent. ALI cultures were conducted as previously described in detail [20, 22]. The stratified squamous epithelial model was ready after 10 days of ALI culture. For monolayer culture, HEECs were cultured in EpiCM-2 in a 96-well plate. Passages 3 to 7 were used for this study.

### Reagents

IFNγ, TNF-α, and IL-33 were purchased from R&D Systems (Minneapolis, MN). JAK inhibitor I (an inhibitor of janus protein tyrosine kinases), SB203580 (a p38 mitogen-activated protein kinase (MAPK) inhibitor), H89 (a protein kinase A (PKA) inhibitor) were purchased from Calbiochem (Milan, Italy). Epigallocatechin gallate (EGCG) [a signal transducer and activator of transcription 1 (STAT1) inhibitor] was purchased from Wako Pure Chemical Industries Ltd.

### Construction of the experimental model and various treatments

In the ALI-culture model, each well has an apical and basal compartment; the apical compartment represents the luminal surface of the esophagus, whereas the basal compartment represents the sub-epithelial surface. Cells were incubated in serum-free medium without bovine pituitary extract for 24 h before stimulation. HEECs were stimulated from the basal compartment by IFNγ (0.1–30 ng/ml), and TNF-α (20 ng/ml). Blocking experiments were performed by pre-incubation with JAK inhibitor I (2 μM), SB203850 (40 μM), H89 (10 μM), and EGCG (20 μM) from the basal compartment for 60 min. IFNγ (30 ng/ml) was then added to the basal compartment in the presence of pretreatment inhibitors. Each experiment was performed in triplicate.

### siRNA

For gene silencing, human IL-33 and STAT1 ON-TARGETplus SMARTpool siRNA (L-015122-01-0005 and L-003543-00-0005, respectively) and a non-specific control siRNA (D-001810-10-05) were purchased from Dharmacon, Inc. (Lafayette, CO). The HEECs in the monolayer model were transfected with 25 nM siRNA using DharmaFECTTM4 (Dharmacon, Inc.). Control and negative siRNA groups were treated with the transfection reagents alone, and with the transfection reagents with non-specific control siRNA, respectively. Cells were incubated for 48 h and the medium was then changed to serum-free basal medium. After 24 h serum-starvation, the medium was changed to fresh basal medium with or without IFNγ (30 ng/ml) and TNF-α (20 ng/ml). The culture supernatants were centrifuged to remove cellular debris and the supernatant was stored at -80°C for further analysis.

### Quantitative RT-PCR (RT-qPCR)

Total mRNA was extracted using Trizol reagent (Invitrogen Life Technologies, Carlsbad, CA), according to the manufacturer’s instructions. cDNA was synthesized using High Capacity cDNA Reverse Transcription (RT) kits (Applied Biosystems, Foster City, CA). Quantitative PCR (qPCR) was carried out using a PCR master mix in a 7900HT Fast real-time PCR System (Applied Biosystems) with TaqMan probes (Applied Biosystems). Primers can be provided upon request.

### Western blot analysis

Cells were collected after stimulation and rinsed with ice-cold PBS/phosphatase inhibitors. Proteins in the total fraction were extracted in a reduced lysis buffer [60 mM Tris-HCL (pH 6.8), 10%glycerol, and protease inhibitor cocktail]. Nuclear extracts were collected according to the instruction of the nuclear extract kit (Active Motif, Carlsbad, CA). Equal quantities of protein were separated by electrophoresis on 10% SDS-PAGE gels and subjected to western blotting with anti-IL-33 (R&D Systems) 1:1000, anti-STAT1 (Cell Signaling, Beverly, MA) 1:1000, Phospho-NF-κB p65 antibody (Cell Signaling) 1:1000 or anti-β-actin (Cell Signaling) 1:1000 overnight at 4°C. After the incubation with the appropriate secondary horseradish peroxidase-conjugated IgG antibody (R&D Systems) for 2 h at RT, the protein bands on the membrane were detected with ECL-Plus Western Blot Detection system (GE Healthcare UK LTD) according to the manufacturer’s instructions. All experiments were replicated at least three times. The results of typical experiments are shown. The western blot bands were analyzed using ImageJ software (Bio-Arts, Co. Ltd, Fukuoka, Japan).

### Cell viability assay

Cell viability was determined using a colorimetric assay based on the cleavage of the tetrazolium salt WST-1 by mitochondrial dehydrogenases (Takara Bio Inc., Otsu, Japan).

### Immunofluorescence staining

The esophageal stratified epithelial layers were fixed in a 2% neutral formalin solution, and embedded in paraffin. Sections (4 μm thick) were cut and deparaffinized using dimethylbenzene followed by dehydration. After antigen retrieval by autoclaving in HistoVT One (Nacalai Tesque, Kyoto, Japan), Protein Block Serum-Free Ready-to-Use (Dako, Carpinteria, CA, USA) was used to minimize non-specific Ig binding. non-specific labeling was blocked with 5% fetal bovine serum/PBS. Slides were then incubated with primary rabbit anti-IL-33 (MBL, Nagoya, Japan) 1:100 or mouse anti-pan Cytokeratin (Abcam, Cambridge, UK) 1:100 overnight at 4°C, Cy3-conjugated goat anti-rabbit IgG (Jackson ImmunoResearch Laboratories, West Grove, PA) and Alexa 488-conjugated goat anti-mouse IgG (Thermo Fisher Scientific Inc. Waltham, MA) secondary antibodies were used at a 1:1000 dilution for 30 min. Nuclear staining was performed using 4^’^,6-diamidino-2-phenylindole (DAPI) (Invitrogen). Slides were viewed using a confocal laser-scanning microscope (FV1000; Olympus).

### Measurement of cytokines

Medium in the basal compartment of the ALI-culture model, and the culture supernatant in the monolayer model were centrifuged to remove cellular debris, then stored at -80°C until analysis. The levels of cytokines and chemokines were measured using a Bio-Plex Human Cytokine 27-Plex panel (Bio-Rad Laboratories), IL-8 ELISA (KHC0081, Invitrogen), and IL-6 ELISA (KHR0061, Invitrogen), according to the manufacturer’s instructions.

### Statistical analysis

All data are presented as the mean ± SD. Data were analyzed using unpaired t-tests for two groups and one-way ANOVA followed by Scheffe's F test for multiple comparisons. All tests were two-sided with a significance level of *P* < 0.05. All authors had access to the study data and have reviewed and approved the final manuscript.

## Results

### IFNγ, but not TNF-α, induces IL-33 mRNA and protein expression in ALI-cultured HEECs

**Fig 1:**
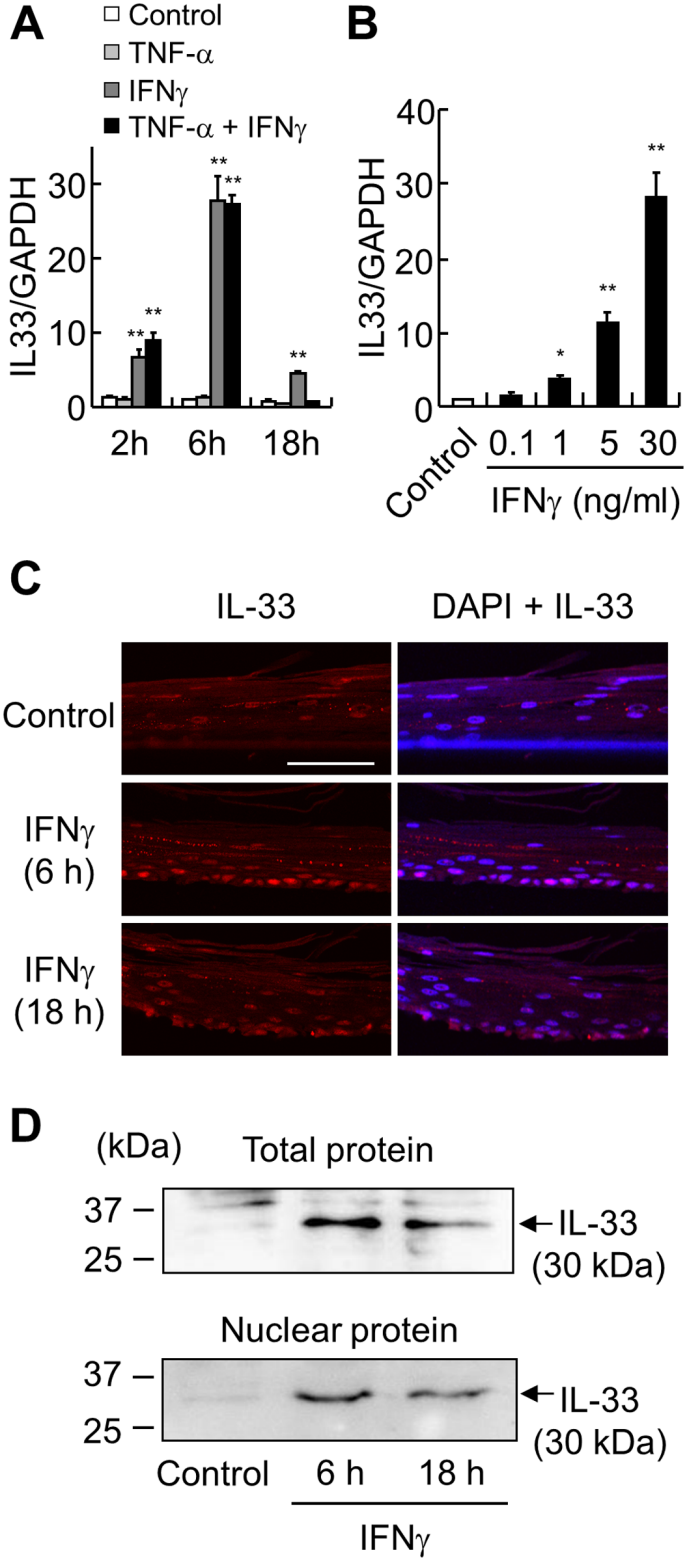
IFNγ, but not TNF-α, upregulates nuclear IL-33. (*A*, *B*) IL-33 mRNA was analyzed by RT-qPCR. (*A*) ALI-cultured HEECs were stimulated with IFNγ (30 ng/ml), TNF-α (20 ng/ml), or both from the basal compartment, and harvested after the indicated time points. (*B*) ALI-cultured HEECs were stimulated with IFNγ (0.1, 1, 5, and 30 ng/ml) and harvested after 6 h. Each value represents the mean ± SD of 3 independent experiments. *P < 0.05, **P < 0.01. (*C*) Immunofluorescence staining of IL-33 (red) in ALI-cultured HEECs after IFNγ (30 ng/ml) stimulation at the indicated time points was performed. DAPI (blue) was used as the nuclear marker. Bar = 50 μm. (*D*) Relative levels of IL-33 in total or nuclear protein extract were assessed by western blot analysis at the indicated time points after IFNγ (30 ng/ml) stimulation.

IFNγ and TNF-α have been reported to induce IL-33 expression in other cell types, such as keratinocytes [16] and airway smooth muscle cells [23]. The expression of IFNγ and TNF-α have also been reported to be increased in GERD patients [2, 8]. Therefore, we investigated the effect of these cytokines on the production of IL-33 in our esophageal stratified squamous epithelial cell model. RT-qPCR revealed that IFNγ, but not TNF-α, significantly increased IL-33 mRNA compared with untreated cells in a time- and dose-dependent manner. Furthermore, the combination of these two cytokines did not further induce IL-33 mRNA (Fig 1*A* and 1*B*). Immunofluorescence staining of IL-33 induced by IFNγ was mainly located in the nucleus of basal layer cells (Fig 1*C*). Western blots confirmed that IFNγ-induced full length IL-33 (30 kDa), and that full length IL-33 was detected in the nuclear fraction of the HEECs (Fig 1*D*).

**Fig 2:**
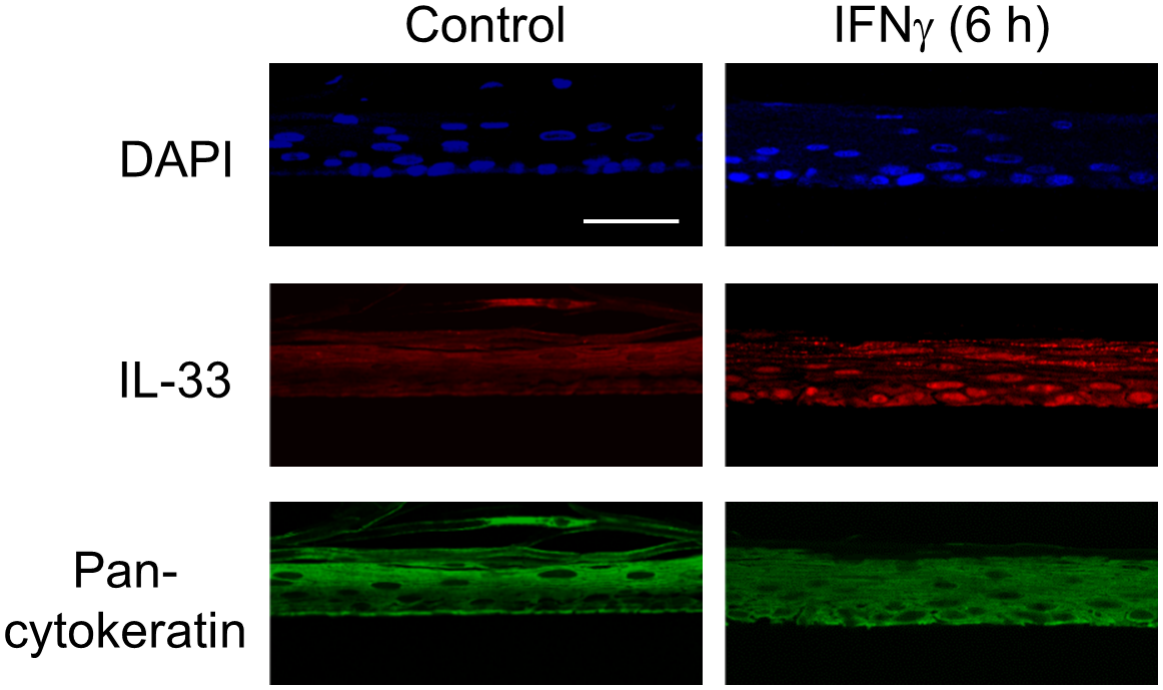
Colocalization of IL-33 and pan-cytokeratin in ALI-cultured HEECs. Immunofluorescence staining of IL-33 (red) and pan-cytokeratin (green) in ALI-cultured HEECs after IFNγ (30 ng/ml, 6 h) stimulation was performed. DAPI (blue) was used as the nuclear marker. Bar = 50 μm.

In order to further confirm the cell type expressing IL-33, the colocalization of immunoreactive IL-33 and an epithelial marker (pan-cytokeratin) was evaluated in control and IFNγ groups, esophageal epithelial layers were positive with pan-cytokeratin at cytoplasmic parts and IL-33 was positive at nuclear (Fig 2).

### Exogenous IL-33 does not induce IL-8 or IL-6 in ALI-cultured HEECs

**Fig 3:**
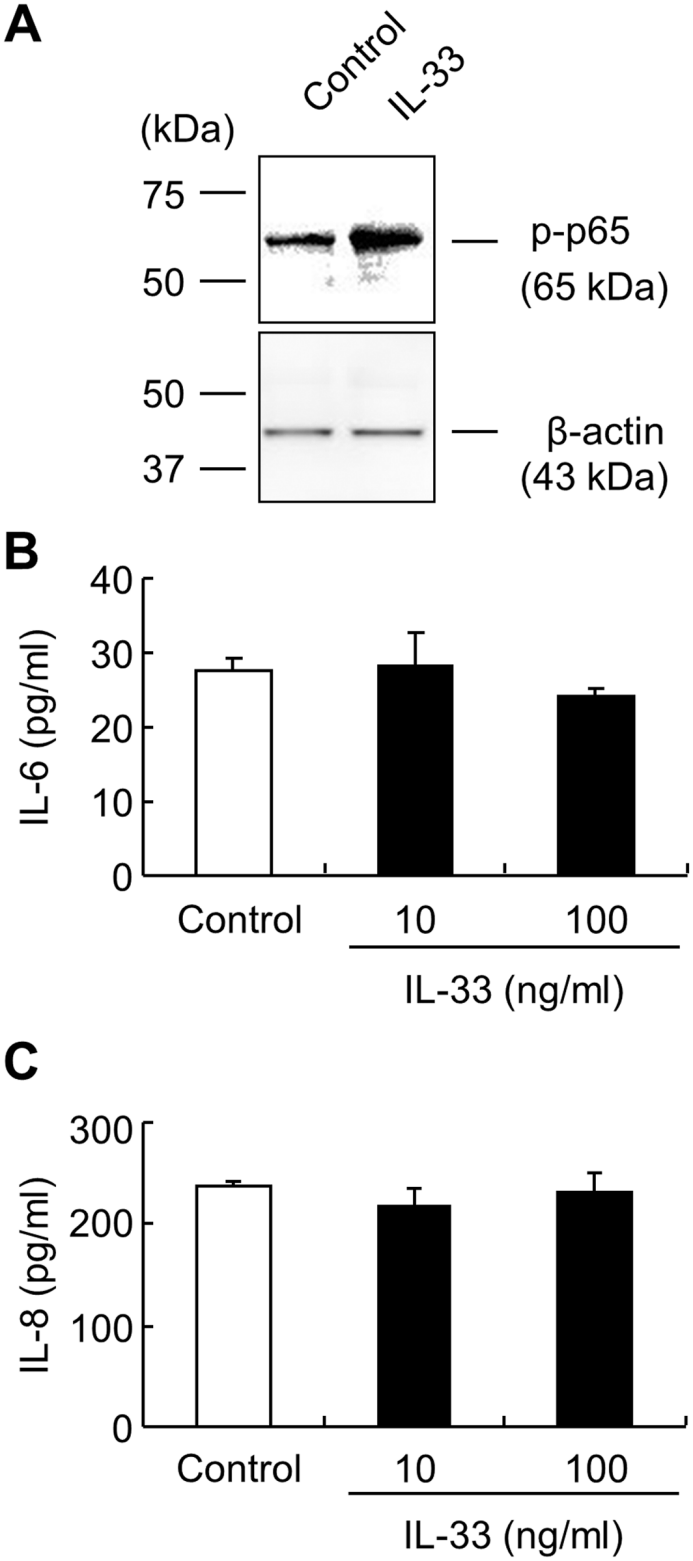
Exogenous IL-33 does not induce cytokines. (*A)* Relative levels of phosphorylated NF-κB p65 (p-p65) were assessed by western blot analysis after IL-33 (50 ng/ml, 1 h) stimulation. β-actin was used as a loading control. (*B*, *C*) ALI-cultured HEECs were stimulated with IL-33 from the basal compartment (10, 100 ng/ml) for 24 h. IL-8 and IL-6 production was determined by ELISA. Each value represents the mean ± SD of 3 independent experiments.

Previous studies indicated that extracellular IL-33 bound the ST2 plasma membrane receptor, thereby activating NF-κB and MAPK to produce inflammatory cytokines such as IL-8 and IL-6 [24]. Although we have confirmed that ST2 is expressed in HEECs (data not shown) and exogenous IL-33 (50 ng/ml, 1h) induced phosphorylation of NF-κB p65 (Fig 3*A*), IL-8 or IL-6 was not induced by exogenous IL-33 (10–100 ng/ml, 24 h) in ALI-cultured HEECs (Fig 3*B* and 3*C*).

### Signaling pathways involved in IFNγ-induced cytokine production

**Fig 4:**
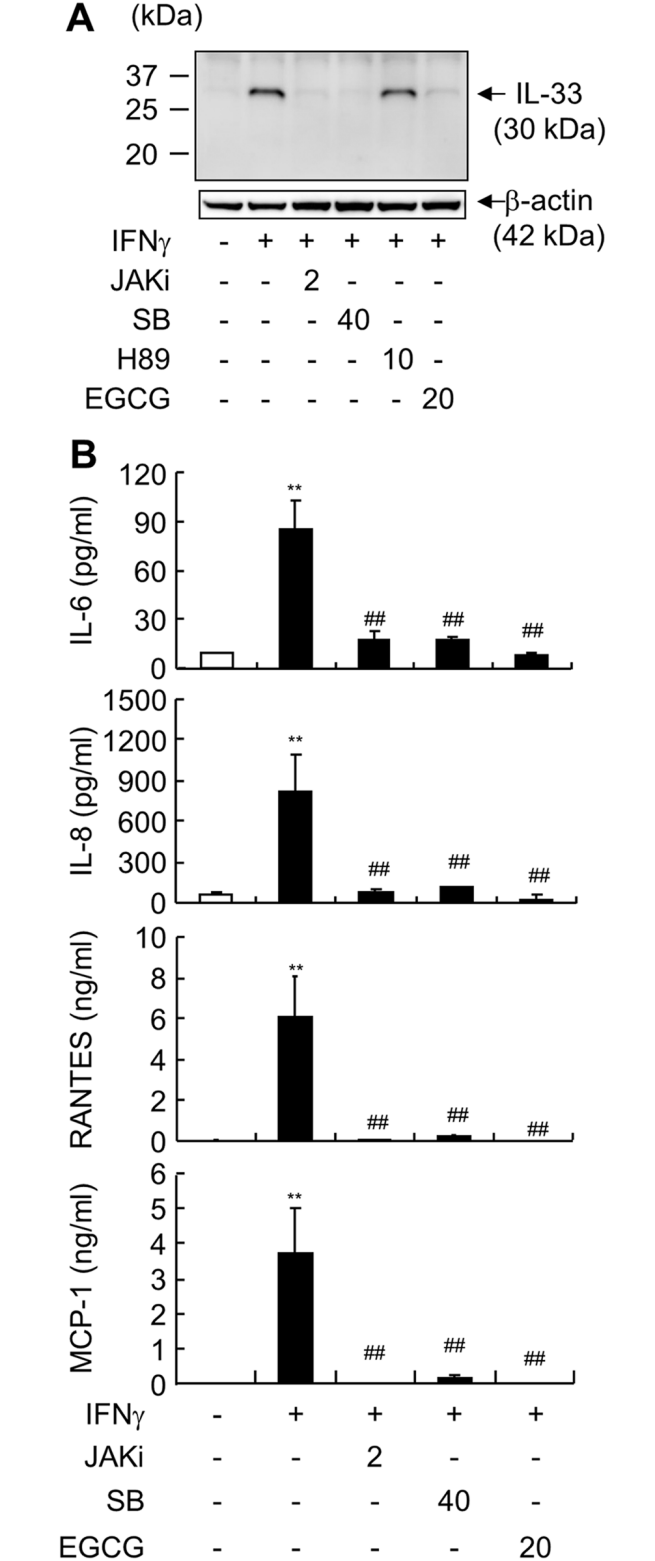
IFNγ-induced cytokine production is p38 MAPK and JAK/STAT1 dependent. ALI-cultured HEECs were pre-incubated with inhibitors of JAKs (2 μM, JAKi), p38 MAPK (40 μM, SB), PKA (10 μM, H89), or STAT1 (20 μM, EGCG) for 1 h, and subsequently co-incubated with IFNγ (30 ng/ml). (*A*) Cells were harvested 10 h after IFNγ stimulation to evaluate IL-33 production by western blot analysis (loading control: β-actin). (*B*) Media from the basal compartment were harvested 24 h after IFNγ stimulation to analyze IL-6, IL-8, RANTES, and MCP-1 production using the Bio-Plex assay. Each value represents the mean ± SD of 3 independent experiments. **P < 0.01 vs. Control. ^##^ *P* < 0.01 vs. IFNγ group.

Previous studies have shown that IFNγ induces the activation of a variety of signaling pathways [25]. To clarify the signal transduction pathways involved in IL-33 expression, we used specific inhibitors of intracellular signaling. IFNγ-induced IL-33 production in ALI-cultured HEECs was almost completely inhibited by JAK inhibitor I, SB203580, and EGCG, but not H89 (Fig 4*A*). Several cytokines and chemokines are elevated in GERD patients, such as IL-8, IL-6, IL-1β, RANTES, and MCP-1 [1, 2]. Using multiplex flow immunoassay, we found IFNγ could induce production of IL-8, IL-6, RANTES, and MCP-1 in ALI-cultured HEECs, and that this effect could be blocked by JAK inhibitor I, SB203580, and EGCG (Fig 4*B*).

**Fig 5:**
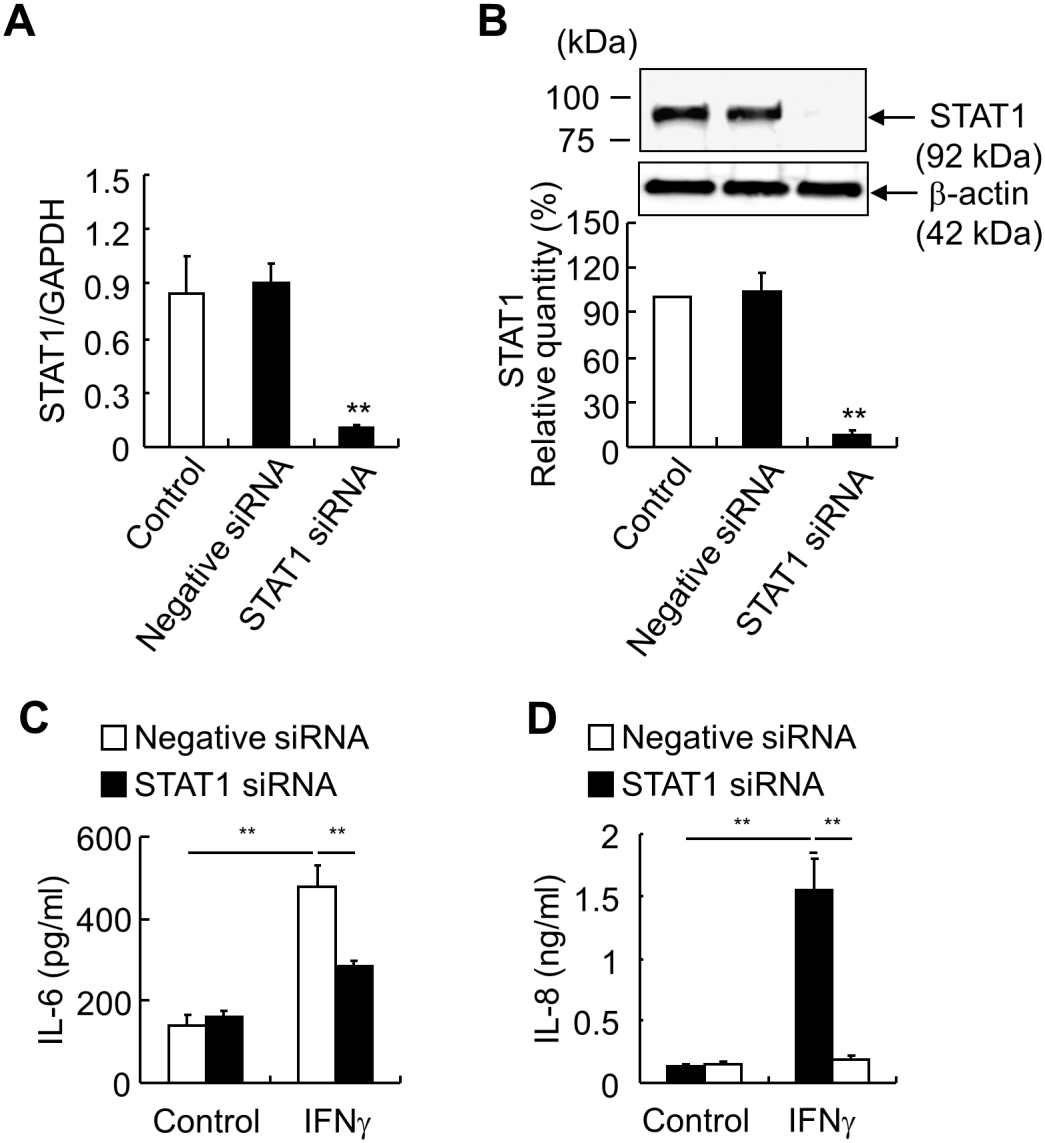
STAT1 knockdown blocks IFNγ-induced IL-6 and IL-8 in monolayer HEECs. STAT1 siRNA and non-specific control siRNA (negative siRNA) were transfected into monolayer HEECs 72 h before stimulation. (*A*) STAT1 expression was evaluated using RT-qPCR after transfection. (*B*) STAT1 production was evaluated by western blot analysis after transfection. (*C*, *D*) The production of IL-6 (*C*) and IL-8 (*D*) 24 h after IFNγ (30 ng/ml) stimulation was detected by ELISA in the supernatants of negative siRNA and IL-33 siRNA treated groups. Each value represents the mean ± SD of 3 independent experiments. **P < 0.01.

To confirm the specificity of EGCG inhibition of STAT1, we transfected HEECs with STAT1 siRNA in monolayer HEECs. STAT1 knockdown was confirmed at both the mRNA and protein levels (Fig 5*A* and 5*B*). IFNγ-induced IL-6 and IL-8 production was almost completely inhibited by STAT1 knockdown (Fig 5*C* and 5*D*).

### Nuclear IL-33 affects IFNγ-induced cytokine production

**Fig 6:**
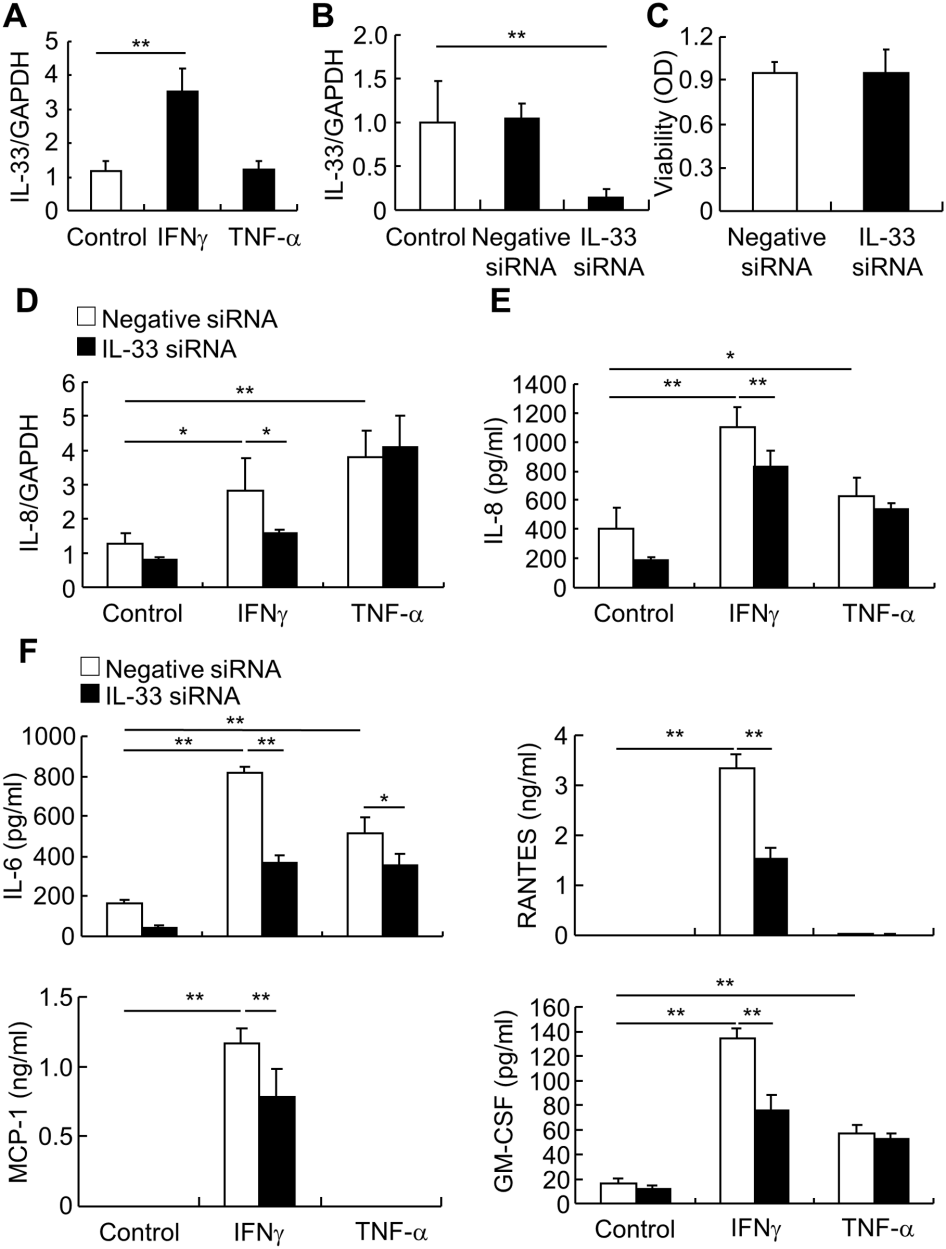
IFNγ, but not TNF-α, -induction of cytokine production is IL-33 dependent. (*A*) Monolayer HEECs were stimulated with IFNγ (30 ng/ml) or TNF-α (20 ng/ml) for 6 h and IL-33 mRNA was subsequently analyzed by RT-qPCR. (*B*) IL-33 siRNA and non-specific control siRNA (negative siRNA) were transfected into monolayer HEECs. IL-33 expression was evaluated by RT-qPCR 72 h after transfection. (*C*) Cell viability 72 h after transfection was examined by WST-1. (*D-F*) After 72 h transfection, monolayer HEECs were stimulated with IFNγ (30 ng/ml) or TNF-α (20 ng/ml). (*D*) IL-8 expression was analyzed by RT-qPCR after 6 h stimulation. (*E*) IL-8 production was analyzed by ELISA after 24 h stimulation. (*F*) In the supernatant of negative siRNA and IL-33 siRNA-treated groups, the production of IL-6, RANTES, MCP-1, and GM-CSF 24 h after IFNγ or TNF-α stimulation were assessed using the Bio-Plex assay. Each value represents the mean ± SD of 3 independent experiments. *P < 0.05, **P < 0.01.

In order to investigate the function of nuclear IL-33, we suppressed its expression with siRNA in monolayer HEECs. After confirming that IFNγ, but not TNF-α, also up-regulated IL-33 mRNA in monolayer HEECs (Fig 6*A*), HEECs were transfected with IL-33 siRNA and IL-33 knockdown was confirmed by RT-qPCR (Fig 6*B*). Cell viability was not affected by IL-33 knockdown (Fig 6*C*). As IFNγ affects the production of IL-33 and several cytokines, we investigated the effect of IL-33 knockdown on the production of IFNγ-induced inflammatory cytokines. The level of IL-8 mRNA and the production of IL-8 were both upregulated by IFNγ and TNF-α, however, IL-33 knockdown only dampened IFNγ-induction of IL-8 (Fig 6*D* and 6*E*). IL-6 production was upregulated by both IFNγ and TNF-α, and was blocked by IL-33 knockdown. Only IFNγ upregulated RANTES and MCP-1 production, which was blocked by IL-33 knockdown. Granulocyte-macrophage colony-stimulating factor (GM-CSF) was upregulated by IFNγ and TNF-α, and IL-33 knockdown only dampened IFNγ-induced GM-CSF (Fig 6*F*).

## Discussion

Inflammatory processes initiated by esophageal epithelial cells are believed to be involved in the pathogenesis of GERD [7]. A group of inflammatory cytokines have been shown to be abundant and released in the GERD mucosa. These included IL-8, IL-6, IFNγ, TNF-α, IL-1β, IL-10, MCP-1, and RANTES [1–3, 8, 9]. A new tissue-derived cytokine, IL-33, has been shown to be upregulated in the nucleus of esophageal epithelial cells in GERD, and takes part in mucosal inflammation [18]. In the present study, we aimed to investigate the production of inflammatory cytokines and their regulation in an *in vitro* three-dimensional esophageal squamous epithelial cell model (ALI-cultured HEECs).

Several studies have demonstrated that IL-33 expression can be upregulated in epithelial, mesenchymal, and myeloid cells cultured with proinflammatory stimuli, such as IFNγ, and TNF-α [16, 17]. As IFNγ and TNF-α levels are increased in GERD [8, 9], we examined their effect on the expression of IL-33 in HEECs. Only IFNγ was found to significantly upregulate IL-33 in esophageal epithelial cells. These data are consistent with reports examining skin [12, 26]. Although a previous study showed the production of the mature form of IL-33 in keratinocytes when stimulated with IFNγ plus TNF-α [16], we did not observe a 20-kDa band when stimulating HEECs with IFNγ plus TNF-α (data not shown). However, we noticed that after 18 h of stimulation with IFNγ plus TNF-α, IL-33 mRNA levels were significantly lower than in the IFNγ-alone group. Although it is not currently clear whether TNF-α attenuated IFNγ-induced IL-33, these data are similar to findings in skin [26]. Therefore, further studies focusing on the mutual effects of IFNγ and TNF-α on the production of IL-33 are warranted.

IL-33 can act as a dual function protein, similar to other IL-1 family cytokines such as IL-1α and IL-37. When released from the cell, the C-terminal IL-1-like cytokine domain of IL-33 can bind to the transmembrane protein ST2, which is an IL-33 receptor. The binding is followed by activation of NF-B and MAPK, thereby resulting in the induction of proinflammatory cytokines and chemokines from immune cells [11] and epithelial cells [16]. As a nuclear protein, its function is still controversial. Ali et al. [12] showed that nuclear IL-33 blocks inflammatory signals, such as NF-κB, in keratinocytes. Conversely, our previous study revealed that nuclear IL-33 has a proinflammatory effect on esophageal epithelial cells [18]. In the present study, IFNγ-induced IL-33 was located in the nucleus of esophageal epithelial cells, and its release from the cells was not detected (data not shown). Although we have confirmed that ST2 is expressed on HEECs (data not shown) and exogenous IL-33 induced phosphorylation of NF-κB p65, IL-8 or IL-6 was not induced by exogenous IL-33 in ALI-cultured HEECs. These data are not consistent with studies performed on keratinocytes [16] and corneal epithelial cells [24]. In these cells, IL-33 acts as a cytokine inducing IL-8 and IL-6 through ST2. This discrepancy might be due to differences in cell type with the function of esophageal epithelial derived IL-33 limited to that of a nuclear factor, unlike other cytokines.

Mucosal inflammation in GERD can result in mucosal disruption, abnormal motility, fibrosis, and carcinogenesis [27]. In patients with GERD, a large number of cytokine and chemokine levels can be increased in mucosal biopsy specimens, including IL-1β, IL-6, IL-8, IL-10, IFNγ, MCP-1, and RANTES [2, 8, 9]. Most of these factors are detected through protein assessment using immunohistochemical staining in tissue lysates, or measurement of mRNA. The sources of these inflammatory mediators are not well defined. In this study, we used a primary human esophageal squamous epithelial cell model. Compared with traditional monolayer cell culture, this model shows similarities with *in vivo* esophageal epithelium, with respect to morphology, molecular marker expression, and barrier function [22]. Furthermore, this model excludes the influence of other cell types, such as neural and immune cells, which may be present in biopsy specimens. The data showed that IL-6, IL-8, RANTES, MCP-1, and GM-CSF are secreted by esophageal epithelial cells and that they are upregulated by IFNγ in an IL-33 dependent manner. Although IL-8 and GM-CSF are also upregulated by TNF-α, IL-33 did not participate in these effects. These data indicate that epithelial cells can release cytokines that are overexpressed in GERD esophageal mucosa, and that these effects are IFNγ and IL-33 dependent.

Previous studies did not find GM-CSF in GERD biopsy specimens. ALI-cultured esophageal cells could secrete GM-CSF, as well as IL-6, IL-8, RANTES, and MCP-1 to the basal side. It is reported that colon epithelial cells can produce GM-CSF, which is required for epithelial cell proliferation and mucosal repair [28]. On the other hand, IL-33 has also been shown to be involved in skin wound healing [29] and colonic mucosal repair [30]. Except for participating in mucosal inflammation, it is unclear whether IL-33 is also involved in mucosal repair in GERD pathogenesis, which is a topic of interest for future studies.

We further investigated the signaling pathways of IFNγ-induced IL-33, IL-6, IL-8, MCP-1, and RANTES. Inhibitors of JAK, p38 MAPK, and STAT1 could completely block IFNγ-induction of IL-33. STAT1 knockdown by siRNA also completely blocked IFNγ-induction of IL-33. These results were consistent with a previous study using keratinocytes [16]. The PKA-CREB pathway has also been shown to be involved in LPS-induction of IL-33 in macrophages [31], and IFNγ can activate PKA and stimulate CREB [32]. However, inhibition of PKA did not affect IFNγ-induction of IL-33. Furthermore, we revealed that the p38 MAPK and JAK/STAT1 pathways are also involved in IFNγ-induced IL-6, IL-8, MCP-1, and RANTES production. Combined with our previous study that IL-33 siRNA blocked IFNγ-induced IL-8 and IL-6 [18], STAT1 activation is in the upstream of IL-33 production. However, the detailed function of nuclear IL-33 is still not clear, further investigations are warranted to explore the nuclear IL-33-induced transcriptional mechanism of these cytokines.

There is some other limitations of the present study. Except for epithelial cells, IL-33 has been shown to be expressed in other cell types, such as endothelial cells [33], fibroblasts [17]. Judd et al. [33] recently showed IL-33 was upregulated in the endothelial cells of eosinophilic esophagitis. However, due to the pure epithelial cell model *in vitro* and superficial biopsy samples, we could not explore the IL-33 expression in other cell types in esophagus. Further studies are required to investigate this part.

In summary, this study revealed that IFNγ upregulates nuclear IL-33 in esophageal epithelial cells. Esophageal epithelial cells can produce various inflammatory cytokines, such as IL-6, IL-8, MCP-1, and RANTES following stimulation by IFNγ. Induction of these cytokines by IFNγ is mediated through the same signaling pathways, namely p38 MAPK and JAK/STAT1. Nuclear IL-33, but not exogenous IL-33, amplified IFN-induced IL-6, IL-8, MCP-1, RANTES, and GM-CSF. This study demonstrates that esophageal epithelial cells can actively participate in GERD pathogenesis through the production of various cytokines, and epithelial cell-derived IL-33 might play a central role in the production of these cytokines.
